# American Med. Association—Next Meeting

**Published:** 1855-03

**Authors:** Francis West

**Affiliations:** 352, Chestnut St., Phila.; One of the Secretaries


### AMERICAN MEDICAL ASSOCIATION.

The eighth annual meeting of the American Medical Association will be held in the city of *Philadelphia*, on Tuesday, May 1, 1855.

The secretaries of all societies, and other bodies entited to representation in the Association, are requested to forward to the undersigned correct lists of their respective delegations *as soon as they may be appointed;* and it is *earnestly* desired by the Committee of Arrangements that the appointments be made at as early a period as possible.

The following are extracts from Article Second of the Constitution:—

"Each local society shall have the privilege of sending to the Association one delegate to every ten of its regular resident members, and one for every additional fraction of more than half this number. The Faculty of every regularly constituted medical college or chartered school of medicine shall have the privilege of sending two delegates. The professional staff of every chartered or municipal hospital containing a hundred patients or more, shall have the privilege of sending two delegates, and every other permanently organized medical institution of good standing shall have the privilege of sending one delegate."

"Delegates representing the medical staffs of the United States Army and Navy shall be appointed by the chiefs of army and navy medical bureaux. The number of delegates so appointed shall be four from the army medical officers."

The latter clause, in relation to delegates from the army and navy, was adopted as an amendment to the Constitution at the meeting of the Association held in New York, in May, 1853.

FRANCIS WEST, M. D., 352, *Chestnut St., Phila.*
*One of the Secretaries.*

